# Acute referral of patients from general practitioners: should the hospital doctor or a nurse receive the call?

**DOI:** 10.1186/1757-7241-19-47

**Published:** 2011-08-11

**Authors:** Christian B Mogensen, Anne Mette M Mortensen, Peter B Staehr

**Affiliations:** 1 Emergency and Acute Admission Department, Kolding Hospital, Denmark

## Abstract

**Background:**

Surprisingly little is known about the most efficient organization of admissions to an emergency hospital. It is important to know, who should be in front when the GP requests an acute admission. The aim of the study was to analyse how experienced ED nurses perform when assessing requests for admissions, compared with hospital physicians.

**Methods:**

Before- and after ED nurse assessment study, in which two cohorts of patients were followed from the time of request for admission until one month later. The first cohort of patients was included by the physicians on duty in October 2008. The admitting physicians were employed in the one of the specialized departments and only received request for admission within their speciality. The second cohort of patients was included by the ED in May 2009. They received all request from the GPs for admission, independent of the speciality in question.

**Results:**

A total of 944 requests for admission were recorded. There was a non-significant trend towards the nurses admitting a smaller fraction of patients than the physicians (68 versus 74%). While the nurses almost never rejected an admission, the physicians did this in 7% of the requests. The nurses redirected 8% of the patients to another hospital, significantly more than the physicians with only 1%. (p < 0.0001). The nurses referred significantly more patients to the correct hospital than the doctors (78% vs. 70% p: 0.03). There were no differences in the frequency of unnecessary admissions between the groups. The self-reported use of time for assessment was twice as long for the physicians as for the nurses. (p < 0.0001).

**Conclusions:**

We found no differences in the frequency of admitted patients or unnecessary admissions, but the nurses redirected significantly more patients to the right hospital according to the catchment area, and used only half the time for the assessment. We find, that nurses, trained for the assignment, are able to handle referrals for emergency admissions, but also advise the subject to be explored in further studies including other assessment models and GP satisfaction.

## Background

Denmark has a well established primary health care system and most of the acutely ill patients are referred to admission by telephone contact between a general practitioner (GP) and a hospital physician on duty; sometimes a consultant specialist, sometimes a newly graduated physician. In a recent study from Denmark it was found, that 87% of all admissions were referrals after direct contact between the GP and a hospital physician [1].

To make best use of funds, personnel and equipment, a correct and timely assessment must be made when the GP contacts the hospital with the intent to admit a patient.

To decide whether a patient should be admitted to hospital is not a trivial matter. It involves professional knowledge of the suspected diagnosis and of the patient's condition to judge whether an admission or another medical service is the best approach. It also requires knowledge of the hospitals ability to handle the patient, which department is capable of delivering the service in need, whether there is an available bed for the patient and whether the patient belongs to the hospitals catchment area. These aspects among others must be considered immediately to avoid delays in the admission of the patient.

Surprisingly little is known about the most efficient organization of admissions to an emergency hospital. In a study from Norway, it was found, that 75% of all requests for admission were handled by doctors and recommended this to be continued [2], whereas another study from the UK in 2007 recommended nurses to be in front on admission and triage [3]. These studies however did not analyse the actual differences in the physicians and nurses ability to assess the patients for admission to an Emergency Department (ED).

The aim of the present study was to analyse how experienced ED nurses perform when assessing requests for acute admissions, compared with hospital physicians, with regard to correct and unnecessary admissions, use of relevant alternatives, time used for the assessment and what happened to the patients, who were not admitted.

## Methods

We conducted a study before and after the introduction of experienced ED nurses to receive the requests for acute admissions.

The Emergency and Acute Admission Department (EAAD) (Akut Modtage Afdeling) at Kolding Hospital was established in august 2008. The EADD is a 36 bed department covering surgical, medical, cardiology, vascular surgery and orthopaedic specialties, and receives around 9.000 admissions annually. In 2008 the physicians employed in each of the specialized departments within the hospital received all telephone requests for admission to their department from the GPs. Beginning in 2009 a specially trained group of nurses in the ED assumed this responsibility.

Only patients referred from a GP or another referring doctor outside the hospital were included in the study, while transfers from other hospitals were excluded.

The first cohort of patients was included by the physicians on duty in October 2008. The admitting physicians were employed in the one of the specialized departments (general surgery, vascular surgery, orthopaedic surgery, internal medicine) and only received request for admission within their own speciality.

The second cohort of patients was included by the ED nurses in the EAAD in May 2009. Around 15 experienced ED nurses were specially trained for this task, with a one-day introduction to the agreements, rules and algorithms for admission and two days supervised practical experience. They received all requests from the GPs for admission, independent of the speciality in question.

For both cohorts the assessing physician or the nurse was asked to note on pre-printed forms the name and ID number of the patient, time of the request, whether the doctor requesting the admission was the patients GP and how much time was used for the assessment.

The physician then had to choose one of the following actions: admission to the EAAD or another department within the hospital, a redirection to a non-urgent admission within the next 24 hours, referral to another hospital, redirection of the patient to a another specialist, referral to an urgent out-patient clinic, referral to a non -urgent out-patient clinic, counselling without admission, or rejection of the request for admission. The chosen strategy was recorded.

The nurses had the same choices, except that a request for admission for vascular surgery or orthopaedic surgery and a request for an urgent out-patient clinic visit in all specialities always was redirected to the relevant specialist.

After discharge the electronic patient record was retrieved and it was recorded whether the patient belonged to the hospital catchment area. For patients who were admitted, the duration of the admission was recorded and whether the admission was considered necessary. An admission was considered necessary if the patient stayed for more than 24 hours, had an advanced radiological examinations (CT- or MR- scan, sonographies, arteriographies), operations or endoscopies performed immediately, received IV treatment, had advanced treatment like DC-conversion, advanced monitoring of vital parameters, including telemetric ECG, or repeated specialist examinations. For the patients where admission was rejected, any admission within the next 30 days was traced and recorded.

All data was entered into a database (Epi-data) and analysed in STATA 7.0. All continuous data were reported as medians and interquartile ranges (IQR), and comparisons were made using the non-parametric Mann-Whitney U-test. All categorical data were reported in absolute numbers and percentage of occurrence and were compared using Fishers exact test or a χ^2 ^test using a 5% confidence level.

The study did not involve any direct contact with the patient and no informed consent or ethical approval was required. The study was registered with the Danish Data Protection Agency (J.nr. 2010-41-5443).

## Results

The physicians included patients to the study from October 1^st ^until October 31st, 2008 and the nurses from May 1^st ^until May 31^st^, 2009. 39 hospital physicians (9 specialists and 30 other non-specialist physicians with 0-5 years experience) and 17 nurses participated in the study. A total of 944 requests for admission were recorded. The baseline characteristics of the two cohorts are shown in table 1.

**Table 1:** Baseline characteristics for the cohorts

Description	Physician Cohort	%	Nurse Cohort	%	p-Value
no. of patients (% of all)	433	(46)	511	(54)	
median age (IQR)	61	(43-75)	59	(39-75)	0.42
gender distribution (M/F) (% male)	188/219	(46)	215/249	(46)	0.97
					
Speciality (% of all)					
					
internal medicine	148	(34)	191	(37)	p < 0.0001
general surgery	150	(35)	204	(40)	
ortopaedic surgery	53	(12)	22	(4)	
vascular surgery	82	(19)	17	(3)	
speciality not defined	0	(0)	77	(15)	
					
Assessment clock time					
					
7 am-3 pm	308	(75)	292	(64)	p < 0.0001
3-pm- 11 pm	73	(18)	153	(34)	
11 pm- 7 am	29	(7)	8	(2)	

While there were no differences in age or gender distribution between the cohorts, there was a significant difference in the distribution between the specialties, mainly because the nurses did not assign 15% of the patients to a speciality before they were seen by a physician. Also more patients were assessed during nighttime by the physicians than by the nurses.

In table 2 the action and outcome after the assessment of the request for an emergency admission is shown.

**Table 2:** Action and outcome of the assessment of patients for emergency admission

Action	physician assessment	%	nurse assessment	%	p-value
redirected to physician/specialist (% of all)	-		83*	(16)	
immediate admissions	315	(74)	350	(68)	0.15
plan for admission within next 24 hours	6	(1)	3	(1)	0.31
redirected to another specialist	8	(2)	21	(4)	0.06
redirected to another hospital	7	(1)	41	(8)	< 0.0001
redirected to the urgent out patient clinic	32	(7)	0	(0)	n.c.
redirected to the non-urgent out patient clinic	36	(8)	12	(2)	< 0.0001
counselling/rejection of admission	24	(7)	1	(0)	n.c.
					
Outcome					
					
unnecessary admissions	18/306	(6)	25/367	(7)	0.62
correct assessment according to catchment area	190/272	(70)	276/355	(78)	0.03
rejected patients admitted within 48 hours**	10/41	(24)	0	(0)	n.c.
median time (minutes) spend on assessment (IQR)***	2	(2-3)	1	(1-2)	< 0.0001

* 51% of these were immediately admitted ** patients rejected or redirected to non-urgent out patient clinic. *** ortopaedic and vascular patients excluded.

While the nurses almost never rejected an admission, the physicians did this in 7% of the requests. The nurses admitted a smaller fraction of patients than the physicians (68 versus 74%) to own hospital and redirected 8% of the patients to another hospital, significantly more than the physicians with only 1%. (p < 0.0001). When controlling the addresses of the patients, the nurses referred significantly more patients to the correct hospital than the doctors (78% vs. 70% p: 0.03).

Among the referrals rejected or redirected to a non-urgent out-patient clinic by the physician, 24% of these patients were admitted within the next 48 hours.

There were no differences in the frequency of unnecessary admissions between the groups. The self-reported median use of time for assessment was 2 minutes for the physicians and 1 minute for the nurses (p < 0.0001). The requests for orthopaedic and vascular admissions were excluded in the time estimate, since these requests always required contact to the hospital specialist.

In 7.7% of the requests the personal ID number was not (correctly) recorded, significantly more frequently in the nurse cohort (11.5% versus 6.3%) than in the physician group (p: 0.005). In the nurse cohort, the contacts resulting in redirection of the GP to another hospital or to a hospital specialists had the highest frequency of missing ID numbers (78% of all missing ID numbers), whereas in the physician cohort it was mainly the requests, resulting in counselling or rejection of request for admission that had information missing (74% of the missing IP numbers).

## Discussion

We found, that there was no difference in the number of admitted patients or in the fraction of unnecessary admissions whether physicians or nurses did the assessment of the GP request for an emergency admission. The nurses redirected significantly more patients to the catchment area hospital, and only used half the time for the assessment. The physicians redirected more patients to outpatient clinics or rejected the request for admission.

When trained staff resources are restricted, it is important to use these in the most effective way. Our findings suggests, that ED nurses, trained for the task, can handle requests for an emergency admission as well as physicians, but differ on certain matters.

This finding seems reasonable. The physicians on duty are not all highly experienced and most are only employed for relatively short periods at the hospital. They might not get familiar with the often complicated rules and routines for admissions or get acquainted with the geographical catchment area of the hospital. In contrast, some nurses have worked for long periods in the emergency field and may have acquired valuable experience assessing the need for an admission as well as a detailed knowledge of the local area and the capability and routines of the hospital.

The hospital doctor made more use of alternatives to admissions than the nurses: non-urgent outpatient clinics (8% vs 2%) and rejection of admissions (7% vs 0%) In 24% of the cases the admission was just postponed in this way by the doctors. In some situations a planned admission in daytime might be a better alternative to an acute admission, but it is difficult to conclude whether it was appropriate or not in this study.

We found that the nurses spend a significant shorter time on assessment for admission but the median difference was only one minute. Although the time saved seems to be minor, it is still time saved both by the admitting physician and the hospital staff. The major advantage, however, might be the reduced numbers of interruptions for the hospital doctor on duty and the time needed to inform the EAAD nurse about the admission.

The nurses still redirected 16% of the request to a physician, which meant an additional contact to another health staff member. Even though almost half of these redirections were orthopaedic or vascular referrals, where this was required according to the algorithms, it still means that a two-step triage was necessary in a considerable number of admissions. It is remarkable, that only in 2% of physician's assessments the admitting doctor was redirected to another specialists. Combined with the observation, that the nurses more often redirected patients to another hospital, it seems that the admitting physician would experience more contacts when the nurse receives the call than the hospital doctor.

In the search for other publications comparing the relative ability of nurses and physicians to assess referrals for emergency admission, we did not find any study similar to our approach or setting. In Norway, the GPs were asked whether they preferred to talk to a nurse or an intern when referring patients. Most GPs preferred to communicate directly with the hospital doctor, bypassing the nurse [4]. In a UK pediatric study, however, the GPs appreciated the assessment from a paediatric nurse [5].

This study has some limitations. Not all referrals were recorded during the study periods, and the distribution between night- and daytime referrals also differs between the two groups, with more inclusions during the evening shift in the nurses group. This might have an effect on possible actions to take. An admission might be a more likely result if the patient was referred in the evening where alternatives like GP consultation or an out-patient clinic is non-existent.

We included the vascular surgery and orthopaedic specialities in the study, to evaluate if the nurses adhered to the decision of redirection these groups to specialists. They did so in all cases. Since the nurses had no options to choose between, it could be argued that these patient groups should not be included. However, it also reflects if the nurses can handle the different algorithms for referrals for different patient groups. So we decided to include these patients in the study, but not in the time estimate where the nurses were merely switch- board operators.

The two cohorts were examined at different times of the year, in October and May. Although this did not include climate extremes there might still be seasonal differences in the admittance rate. An influence on the assessment of patients for admission due to this cannot be excluded. The bed occupancy, which changes with the time of the year might influence the tendency to accept, reject or redirect an admission.

The lack of personal ID numbers in around 8% of the requests did not affect the record of the action taken, but it was not possible to subsequently classify the outcome. The physicians did not record around 4% of the rejected admissions, so readmission rates might be higher or lower than measured.

The definition of an unnecessary admission is debatable, and other authors have used for instance the Appropriateness Evaluation Protocol (AEP). However, the aim was to look for differences in admission patterns, and we used the same criteria for the nurse and the physician group and observed no differences.

In around 15% of the referrals, the nurses admitted a patient without defining the speciality, to which the patient belonged. This was not the setting for the physician since the speciality was already chosen by the GP when he contacted the physician within that speciality. However, not choosing a speciality in a small amount of cases might well reflect a more realistic approach to the patient, since it is often not possible to relate symptoms to a speciality, until the patient has been examined carefully.

Since this study is apparently the first to report quantitative data on the important question of the referral strategy in emergency hospitals, and since our data are incomplete and possibly biased due to differences in the study groups, we suggest further studies to be performed on the subject. Another option is to assign an experienced physician, employed in the EAAD to the task of receiving the call from the GP. This would add medical experience to the knowledge about local geography and rules for admissions and might utilize the out-patient clinics better. However, as long as the physicians are a limited resource in the EAAD, it is necessary to evaluate how the physicians are best used, in the phone or at the patient. We suggest to address this important question in future studies. To avoid some of the above mentioned limitations of the present study a randomized study would be preferable, which could also include an assessment of GP satisfaction with the two approaches for admission.

## Conclusions

We studied how experienced ED nurses assessed the referrals for admission compared with hospital physicians. We found no differences in the frequency of admitted patients or unnecessary admissions, but the nurses redirected significantly more patients to the right hospital according to the catchment area, and used only half the time for the assessment, whereas the physicians rejected more patients or referred to outpatient clinics. We find, that nurses, trained for the assignment, are able to handle referrals for emergency admission.

## Competing interests

The authors declare that they have no competing interests.

## Authors' contributions

CBM concepted the idea for the study and the design. AMMM participated with CBM in the acquisition of data, which was analyzed by CBM and interpreted by all three authors. CBM drafted the manuscript which was revised by PBS and ANMM. All three authors have given final approval of the version to be published.
